# *Notes from the Field*: Detection of Vaccine-Derived Poliovirus Type 2 in Wastewater — Five European Countries, September–December 2024

**DOI:** 10.15585/mmwr.mm7407a4

**Published:** 2025-03-06

**Authors:** Shahin Huseynov, Eugene Saxentoff, Sabine Diedrich, Javier Martin, Magdalena Wieczorek, Maria Cabrerizo, Soile Blomqvist, Jaume Jorba, José Hagan

**Affiliations:** ^1^World Health Organization Regional Office for Europe, Copenhagen, Denmark; ^2^Robert Koch Institute, Berlin, Germany; ^3^Medicines and Healthcare Products Regulatory Agency, Potters Bar, United Kingdom; ^4^National Institute of Public Health NIH - National Institute of Research, Warsaw, Poland; ^5^Instituto de Salud Carlos III, Madrid, Spain; ^6^Finnish Institute for Health and Welfare, Helsinki, Finland; ^7^Division of Viral Diseases, National Center for Immunization and Respiratory Diseases, CDC; ^8^Global Immunization Division, Center for Global Health, CDC.

**Summary**

**What is already known about this topic?**

A vaccine-derived poliovirus type 2 (VDPV2) lineage that originated in Nigeria has been detected in 21 other countries on the African continent.

**What is added by this report?**

During the weeks ending September 22—December 22, 2024, VDPVs genetically linked to the Nigeria lineage were detected in wastewater samples in 16 cities in five European countries. No human polio cases or poliovirus infections were reported in association with these detections.

**What are the implications for public health practice?**

Isolations of VDPV2 from wastewater appear to represent importations of the virus into these countries. Continued circulation of VDPV2 in African countries could result in similar importations and potential transmission in susceptible populations outside of Africa. High coverage with poliovirus vaccines is critical to protect against polio disease and prevent establishment of local circulation following poliovirus importation.

Poliovirus is highly contagious and can cause flu-like illness and in rare cases, acute flaccid paralysis (AFP) and, since 1988, has been targeted by all countries for eradication. Vaccine-derived polioviruses (VDPVs) are rare pathogenic strains of poliovirus that can emerge through genetic mutations in the oral polio vaccine strain, from prolonged circulation in underimmunized populations (*1*). The World Health Organization (WHO) European Region has been free from endemic polio since 2002, but importation of VDPVs from other settings can lead to local circulating VDPVs (cVDPVs) if they are introduced into undervaccinated communities.* Circulation of type 2 cVDPV (cVDPV2) occurred in the United Kingdom in 2022, linked to transmission in Israel, United States, and Canada.^†^ Because most polio infections are asymptomatic and polioviruses are shed in stool, many countries in Europe supplement syndromic AFP and enterovirus surveillance by conducting environmental surveillance (ES) for polioviruses through the systematic sampling of wastewater. During weeks ending September 22–December 22, 2024 (as of January 24, 2025), type 2 VDPV (VDPV2) was detected in wastewater samples collected in five European countries (Spain, Poland, Germany, the United Kingdom, and Finland) with high national coverage with inactivated poliovirus vaccine (IPV). 

## Investigations and Outcomes

The first European detection of VDPV2 was in wastewater sampled in the Barcelona, Spain metropolitan area during the week of September 16–22, 2024. During weeks ending October 27–December 22, 2024, ES samples from 16 total additional sites in metropolitan areas of Poland (two sites), Germany (nine), the United Kingdom (four) and Finland (one) tested positive for related polioviruses (Table). Genetic sequence analysis confirmed that these detections were linked (*1*) to the cVDPV2 NIE-ZAS-1 emergence^§^ that was first detected in Zamfara, Nigeria in July 2020 (*2*), and which continues to circulate in Nigeria in 2024. Spread of cVDPV2 from this emergence group has caused outbreaks of poliomyelitis in 15 other countries in North and West Africa (WHO, unpublished data, 2024) and has been detected by ES in an additional six countries on the African continent (*3*). This activity was reviewed by CDC, deemed not research, and was conducted consistent with applicable federal law and CDC policy.^¶^

**TABLE:** Number of wastewater detections of vaccine-derived poliovirus type 2, by date and location (N = 34) — five European countries, weeks ending September 22–December 29, 2024*

Country/City (no. of detections)	Week ending														
	Sep 22	Sep 29	Oct 6	Oct 13	Oct 20	Oct 27	Nov 3	Nov 10	Nov 17	Nov 24	Dec 1	Dec 8	Dec 15	Dec 22	Dec 29
**Spain (1)**															
Barcelona	X^†^	—	—	—	—	—	—	—	—	—	—	—	—	—	—
**Poland (2)**															
Warsaw	—	—	—	—	—	X	—	—	—	—	—	—	—	—	—
Rzeszów	—	—	—	—	—	—	—	—	—	—	—	X	—	—	—
**Germany (24)**															
Munich	—	—	—	—	—	—	X	—	X	X	—	X	—	—	—
Berlin	—	—	—	—	—	—	—	X	—	—	—	—	—	—	—
Hamburg	—	—	—	—	—	—	—	—	X	—	—	—	—	—	—
Bonn	—	—	—	—	—	—	—	—	X	X	X	X	—	X	—
Cologne	—	—	—	—	—	—	—	—	X	X	X	X	—	X	—
Dresden	—	—	—	—	—	—	—	—	X	—	—	—	—	X	—
Mainz	—	—	—	—	—	—	—	—	X	X	X	—	—	—	—
Düsseldorf	—	—	—	—	—	—	—	—	—	X	—	—	—	—	—
Stuttgart	—	—	—	—	—	—	—	—	—	—	X	X	—	—	—
**United Kingdom (6)**															
Leeds	—	—	—	—	—	—	—	X	—	X	—	—	—	—	—
London (Beckton)	—	—	—	—	—	—	—	—	X	—	—	—	X	—	—
London (Crossness)	—	—	—	—	—	—	—	—	X	—	—	—	—	—	—
Worthing	—	—	—	—	—	—	—	—	—	X	—	—	—	—	—
**Finland (1)**															
Tampere	—	—	—	—	—	—	—	—	—	X	—	—	—	—	—

* Data reported to the World Health Organization as of January 24, 2025.

^†^ “X” indicates weeks in which vaccine-derived poliovirus type 2 was detected in a given sampling location. One detection indicates one week in which poliovirus was detected in a given site.

Sequencing of the European cVDPV2 isolates identified a divergence in the VP1 capsid protein coding region of 43–50 nucleotides from the Sabin 2 vaccine strain. Overall, 38 of these nucleotide differences were found in all European isolates; they have a common divergence of 13 nucleotide changes from the closest known NIE-ZAS-1 isolates, which were previously detected in Algeria, Guinea, and Mali. The group of viruses detected in these European countries represents a single lineage (i.e., they exhibit a common pattern of nucleotide changes that make them more closely related to each other than to any other non-European isolates from the NIE-ZAS-1 emergence); however, there is a range of genetic differences within the cluster, and contemporaneous isolations from different sites in the same country exhibited substantial divergence from one another (*4*). 

Importantly, no human polio cases or asymptomatic infections were reported in association with detection of this group of viruses.

## Preliminary Conclusions and Actions

Since circulating polioviruses are known to accumulate mutations at an average of about one nucleotide change per month, (*5*) the common 13-nucleotide difference from the parent cluster suggests approximately 1 year of undetected circulation outside the catchment areas of the European ES network before this detection event. To date, the data (i.e., the diversity of isolates within the same geographic area and the paucity of repeated isolations of closely related virus from a single site) suggest that these importations have not resulted in substantial person-to-person transmission. The affected countries are actively working to prevent establishment of local transmission by vaccinating targeted populations with IPV to close immunity gaps and they have intensified AFP surveillance and ES for poliovirus. This event highlights the ongoing risk from circulating VDPVs worldwide and the need for intensified global efforts to eradicate all polioviruses. Wastewater surveillance is a useful supplement to high quality AFP surveillance to detect importation and monitor potential population risk. High coverage with poliovirus vaccine across all population groups is critical to protecting against AFP and other forms of polio disease and preventing establishment of local circulation following poliovirus importation. 

## Footnotes

* The target immunization coverage for polio is ≥90% of children receiving 3 doses of polio-containing vaccine by age 1 year.

† https://www.who.int/emergencies/disease-outbreak-news/item/2022-DON408

§ Emergences of poliovirus are designated by the country and geographic subnational region of the emergence and the number of emergences in each subnational region.

¶ 45 C.F.R. part 46.102(l)(2), 21 C.F.R. part 56; 42 U.S.C. Sect. 241(d); 5 U.S.C. Sect. 552a; 44 U.S.C. Sect. 3501 et seq.
